# Targeting CD38 in Neoplasms and Non-Cancer Diseases

**DOI:** 10.3390/cancers14174169

**Published:** 2022-08-28

**Authors:** Wojciech Szlasa, Jakub Czarny, Natalia Sauer, Katarzyna Rakoczy, Natalia Szymańska, Jakub Stecko, Maksymilian Kołodziej, Maciej Kaźmierczak, Ewa Barg

**Affiliations:** 1 Faculty of Medicine, Wroclaw Medical University, 50-367 Wroclaw, Poland; 2 Faculty of Medicine, Poznań University of Medical Sciences, 61-701 Poznań, Poland; 3 Faculty of Pharmacy, Wroclaw Medical University, 50-367 Wroclaw, Poland; 4 Department of Hematology and Bone Marrow Transplantation, Poznań University of Medical Sciences, 61-701 Poznań, Poland; 5 Department of Basic Medical Sciences, Faculty of Pharmacy, Wroclaw Medical University, 50-556 Wrocław, Poland

**Keywords:** CD38, multiple myeloma, anticancer therapy, cancer

## Abstract

**Simple Summary:**

CD38 remains an interesting target for anticancer therapy. Its relatively high abundance in neoplasms and crucial impact on NAD+/cADPR metabolism and the activity of T cells allows for changing the immune response in autoimmune diseases, neoplasms, and finally the induction of cell death. Antibody-dependent cell cytotoxicity is responsible for cell death induced by targeting the tumor with anti-CD38 antibodies, such as daratumumab. A wide range of laboratory experiments and clinical trials show an especially promising role of anti-CD38 therapy against multiple myeloma, NK cell lymphomas, and CD19- B-cell malignancies. More studies are required to include more diseases in the therapeutic protocols involving the modulation of CD38 activity.

**Abstract:**

CD38 is a myeloid antigen present both on the cell membrane and in the intracellular compartment of the cell. Its occurrence is often enhanced in cancer cells, thus making it a potential target in anticancer therapy. Daratumumab and isatuximab already received FDA approval, and novel agents such as MOR202, TAK079 and TNB-738 undergo clinical trials. Also, novel therapeutics such as SAR442085 aim to outrank the older antibodies against CD38. Multiple myeloma and immunoglobulin light-chain amyloidosis may be effectively treated with anti-CD38 immunotherapy. Its role in other hematological malignancies is also important concerning both diagnostic process and potential treatment in the future. Aside from the hematological malignancies, CD38 remains a potential target in gastrointestinal, neurological and pulmonary system disorders. Due to the strong interaction of CD38 with TCR and CD16 on T cells, it may also serve as the biomarker in transplant rejection in renal transplant patients. Besides, CD38 finds its role outside oncology in systemic lupus erythematosus and collagen-induced arthritis. CD38 plays an important role in viral infections, including AIDS and COVID-19. Most of the undergoing clinical trials focus on the use of anti-CD38 antibodies in the therapy of multiple myeloma, CD19- B-cell malignancies, and NK cell lymphomas. This review focuses on targeting CD38 in cancer and non-cancerous diseases using antibodies, cell-based therapies and CD38 inhibitors. We also provide a summary of current clinical trials targeting CD38.

## 1. CD38 Structure and Function

**Figure 1:**
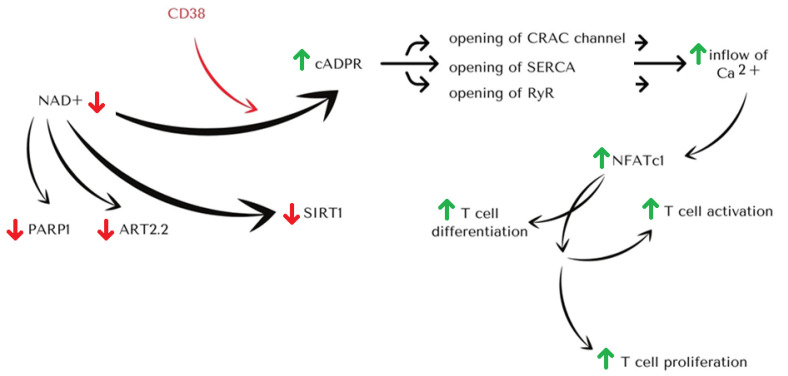
Schematic representation of the role of CD38 in NAD+ metabolism. CD38 depletes NAD+ cellular level, generating cyclic ADP-ribose (cADPR). The process leads to the inflow of Ca^2+^ stimulates T cell activation and proliferation. Depletion of NAD+ level affects the activity of Sirt1, ART2.2, and PARP1, which are NAD+ consuming enzymes that play significant roles in T cell fate determination.

CD38 (cluster of differentiation 38) is a transmembrane glycoprotein of 300 amino acids encoded by a CD38 gene located on chromosome 4 (Figure 1). The molecule may be found both on the plasma membrane and endoplasmic membranous system [1,2]. CD38 is interacting with the B-cell receptor (BCR) in B-cells. Afterward, the cell becomes activated and thus the molecule is a marker of recently activated CLL B-cells [3,4]. The 42 kDa protein is present in various human cells, including NK, CD8+ and CD4+ cells [5,6,7]. The bifunctional enzyme is involved in both the hydrolysis and biosynthesis of cyclic ADP-ribose (cADPR). Mutation studies unraveled that 119 and 201 residues of CD38 are involved in the hydrolytic activity of the enzyme [8]. Activation of the enzyme leads to the increase of the intracellular Ca^2+^ concentration by its mobilization from the endoplasmic reticulum via RyR receptors [9]. Interestingly, the catalytic domain of CD38 remains extracellular, thus the mechanism in which it activates the T-lymphocytes remains unclear. Some authors suggest that the internalization of the enzyme or the cADPR may be responsible for the intracellular effects of the protein [10,11]. Another possibility includes the dualistic role of the receptor depending on its cellular localization. Namely, the extracellular enzymatic activity is involved in the degradation of nicotinamide mononucleotide (NMN) for nicotinamide adenine dinucleotide (NAD+) synthesis. CD38 turns out as a key regulator of immune checkpoints involving NAD+.

CD38 might also be involved in cell migration and proliferation via the interactions with the CD31 molecule. Studies by Deagilo et al. [12], proved that the interaction between both antigens induces the proliferation of CLL lymphocytes and promotes the localization of leukemic cells in the growth-permissive sites.

The enzyme is responsible for the biosynthesis of cADPR from NAD+. Besides, it produces nicotinic acid adenine dinucleotide phosphate (NAADP) from NADP [13]. When CD38 remains active, the level of NAD+ decreases, affecting the activity of Poly-ADP-ribosyltransferase 1 (PARP1), ADP-ribosyltransferase 2.2 (ART2.2), and Sirtuin 1 (SIRT1). PARP1 is responsible for the inhibition of Treg suppressive activity, Th2 cells response, and evocation of Th1 cells response. ART2.2 regulates T cell apoptosis and early signaling events in T cell activation. Finally, SIRT1 regulates T cell metabolism and epigenetic conjuncture. On the other hand, the increase in cADPR concentration leads to the opening of the Ca^2+^ release-activated Ca^2+^ channel (CRAC), Sarco(endo)plasmic reticulum Ca^2+^-ATPase (SERCA), and Ryanodine receptors (RyR) channels, leading to the increase in Ca^2+^ concentration. Calcium ions activate the NF-AT factor, which results in T cell activation, differentiation, and proliferation.

Its role in cell migration, apoptosis, and cytokine release was reported [1,14,15,16,17,18]. Also, CD38 was first described as a surface protein in T cells that can induce cell activation [2,18]. The pattern of CD38 expression during the cells’ life cycle suggests that CD38 significantly influences a wide range of processes. At the molecular level, its enzymatic activity is connected with the pathogenesis of aging, diabetes, obesity, asthma, and inflammation. Moreover, CD38 acts as a cell surface marker in hematologic malignancies such as multiple myeloma, the disease in the therapy of which cytotoxic anti-CD38 antibodies are used. However, due to the multitude of its functions, there are many more potential therapeutical deployments targeting CD38 [19]. The review presents a comprehensive description of CD38’s application in the anticancer therapy and treatment of non-cancer diseases.

## 2. CD38 Expression

CD38 is widely expressed in various human tissues including prostatic epithelial cells, pancreatic islet astrocytes, smooth muscle cells, retinal tubes, kidneys, gut, brain, and immune system cells [2]. The abundance decreases from plasma cells, natural killer (NK cells), B lymphocytes, dendritic cells, LiT cells, hematopoietic stem cells, up to monocytes [20,21]. Interestingly CD38 has not been found in any fetal organ or tissue yet [22]. It is widely spread among hematological neoplasms, e.g., multiple myeloma as well [2,23]. CD38 can also be detected in non-hematopoietic tissues: prostatic epithelial cells, pancreatic islet astrocytes, perikarya, neurons, airway striated muscle cells, renal tubules, and retinal ganglia cells [20].

**Figure 2:**
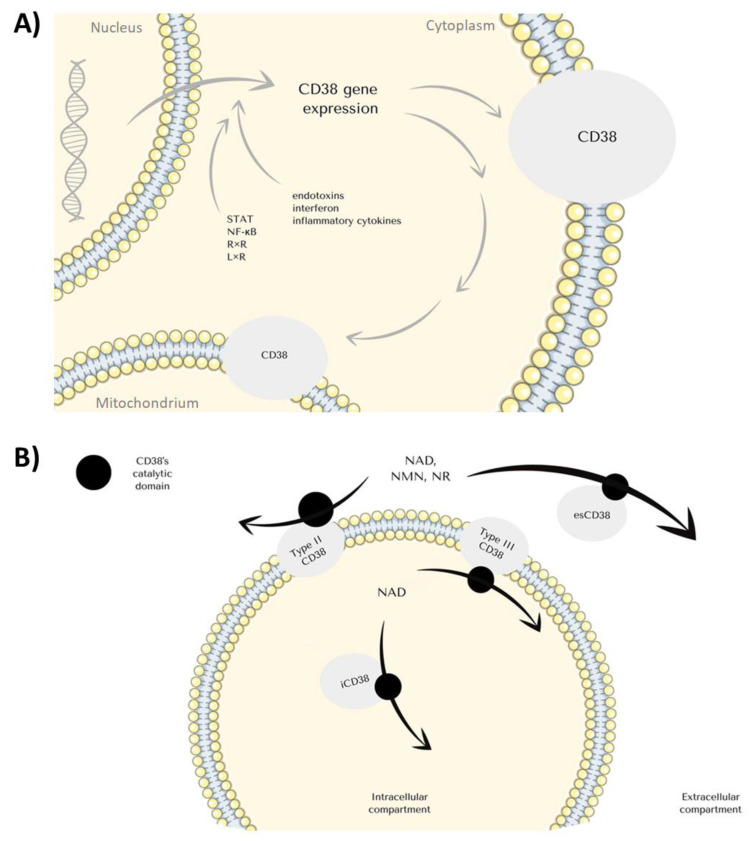
(**A**) Schematic representation of the factors that regulate the expression of the CD38 gene. It is worth noting that CD38 is localized both on the cell membrane and endoplasmic membranous system; (**B**) Schematic representation of the variety of CD38 types with their localization; STAT—signal transducer and activator of transcription, NF-κB—Nuclear factor kappa B, RxR—retinoid X receptor, LxR—Liver X receptor, NAD—Nicotinamide adenine dinucleotide, NMN—Nicotinamide mononucleotide, NR—nicotinamide riboside, ecCD38—extracellular CD38, iCD38—intracellular CD38.

CD38 can be found both on the cell surface and in intracellular compartments, most commonly mitochondria, endoplasmic reticulum, and nuclear membrane (Figure 2B) [19,21,24,25]. The molecule constitutes a single chain glycoprotein with a single transmembrane segment. Depending on the orientation, CD38’s C-terminal catalytic site may face the extracellular environment, or the domain is directed towards the cytosol. A secretory soluble form of CD38 with extracellular enzyme activity has been reported since substrates and products of CD38-catalyzed reactions were generated either in the intracellular or extracellular compartment [25,26,27]. As the intracellular environment constitutes the main source of substrates for CD38’s enzymatic activity, the dominance of its ectoenzymatic structural type might seem paradoxical. Even though NAD+ is the main substrate for the glycoprotein, NMN and NR, circulating NAD+ precursors, can also be degraded by the enzyme (Figure 1) [28,29]. CD38 occurs in various forms, including an intracellular iCD38, extracellular esCD38 and standard membrane form (Figure 2B) [25,26,27]. CD38 plays a key role in the depletion of intracellular NAD levels. The study by Chini et al. shows that inhibition of the eco-enzymatic activity of CD38 leads to a decrease in the level of ADPR—the product of NAD+ degradation [30]. The reason for this may be the metabolism of NMN- (NAD+ precursor) [31] in the extracellular space by CD38 activity [30,32]. CD38 molecule is involved in every part of the inflammation process including migration, aggregation, adhesion, phagocytosis, antigen presentation, and antigen release [20].

Regarding the mechanisms underlying the regulation of CD38 expression, its level remains elevated when macrophages are stimulated with LPS. The promoter region of the CD38 gene is regulated by factors such as signal transducer and activator of transcription (STAT), nuclear factor kappa B (NF-κB), and retinoid X receptor (RxR) and liver X receptors (LxR). Besides, immune response, early phases of haematopoietic differentiation, rheumatoid diseases—systemic lupus erythematosus (SLE), rheumatoid arthritis (RA), loss of antioxidant potential—also lead to the increased expression of CD38 [2,14,20,33]. Differentiation of immune cells is correlated with CD38 expression. It is highly expressed in the B-cells from the germinal center and later, after the maturation, they lose the surface expression [14,20]. Early T cells precursors and thymocytes act likewise. Both T and B-cells upregulate CD38 expression upon activation, and NK seems to express it on a constant level [2,14,20]. CD38 is associated with TCR, CD16, and BCR molecules in T and B lymphocytes, cooperating in transducing activating signals in immune cell populations [14,34,35,36]. Interestingly, CD38+ T lymphocytes can suppress proliferation of the CD38- ones, preventing diabetes type 1 during Mycobacterium avium infection [34,37]. CD38hiCD8+ are capable of suppressing CD4+ proliferation [34].

### 2.1. The Biological Consequences of CD38 Stimulation End Expression

Expression of the CD38 gene can be induced by endotoxins, interferon, and inflammatory cytokines (Figure 2A) [38,39,40]. IFN type I α and β, IFN type II γ are known as pro-stimulatory cytokines, involved in inflammation and interestingly have their binding site on the CD38 promoter. Stimulating leukemic B-cells with interferon results in induction of CD38 mRNA synthesis [41], therefore there is a direct connotation between CD38 and inflammation. When CD38 activity is induced, the Ca^2+^ level elevates rapidly, evolving in the migration of leukocytes through the endothelium, the effect possibly correlated with CD38’s ligand-CD31 or interacting with hyaluronic acid [2,41,42]. After migration chemotaxis to the site of injury occurs, and it does when chemokine receptors CXCR4, CCR7, and FPR1 are present. Ca^2+^ peak plays a role in inducing expression of those [20,43]. CD38 is required in TFEB (transcription factor for EB, the primary transcription factor involved in regulating the expression of lysosomal elements) activation by ROS (reactive oxygen species) during infection [44]. TFEB is a contributor to phagocytosis in macrophages; this pathway contributes to several interactions between these chemokines, resulting in TFEB nuclear import and gene induction afterward [44]. Also, mice microglia devoid of CD38 exhibit lower phagocytic activity [45,46]. Superantigens are microbial proteins, potent stimulators of the immune cells unconventionally [47]. Ligation of CD38 inhibits LiT proliferation induced by superantigens, and Zilber et al. show that CD38 and MHC class II share a common activation pathway [47]. Their experiments indicate that CD38 with MHC II displays lateral associations on monocytes, and both play a role in the transduction of signals after superantigen activation and co-work in antigen presentation [24,47]. In another work, they also point out that CD38/CD9 blocking inhibits LiT response [48]. Lische et al. infected knockout mice with Listeria monocytogenes and afterward the absence of CD38 caused alteration in the recruitment of neutrophils and inflammatory monocytes, still preserving their ability to kill the intracellular pathogens [49]. The absence of CD38 on T cells leads to poor humoral immune response, which results in vulnerability to extracellular pathogens such as Streptococcus pneumoniae [43]. Intracellular NADase activity leads to Ca^2+^ mobilization due to cADPR production [2]. Failure of this mechanism in CD38- dendritic cells causes the inability to migrate in response to chemokines and priming of T cells [43].

### 2.2. CD38 Expression and Inflammation

CD38 and inflammation exist in relation to each other. Nevertheless, it is not clear when it comes to chronic inflammation diseases. Their pathogenesis used to be linked to deviation from the Th1/Th2 axis, however, it is not as straightforward as we thought and the theory itself has its limitations [50]. CD38 expression is elevated in colon specimens from patients with inflammatory bowel disease (CD or UC) compared to noninflamed ones, and also it is expressed more in inflamed regions than in healthy regions [33]. Tissues of rheumatoid arthritis (RA) patients exhibit elevations in CD38, too [51]. A high proportion of CD38+ NK cells are exhibited in the blood and fluids of RA patients, possibly disturbing immune tolerance, as an outcome of inhibiting Treg differentiation [52]. Ning et al. suggest potential therapy of RA and lupus erythematosus (SLE) through inhibition of CD38 [33,53]. Other authors seem to disagree with this approach. Pérez-Lara et al. believe that CD38+ Tregs are more suppressive than CD38-Treg, even suggesting that overexpressing CD38 in SLE patients could prevent inflammation instead of enhancing it [54]. This is due to the correlation between CD38 with FoxP3 and downstream production of immunosuppressive cytokines: D69, IL-10, CTLA-4, and PD-1 [54]. Some SLE patients suffer from frequent infections; they also have increased CD38hi LiT, which probably is the reason for increased vulnerability to infections [55]. The mechanism consists of acetylation of EZH2, which represses RUNX3 expression, and the cytotoxic LiT response is reversed when EZH2 action is inhibited [55]. Every action of CD38 seems to be associated with Ca^2+^ release. CD38 facilitates pathways that lead to the synthesis of pro-inflammatory cytokines and the suppressive ones simultaneously. More authors however tend to look in the pro-inflammatory direction as the potential side of interest or treatment.

## 3. Anti-CD38 Therapeutics

Specific cancer cells, such as multiple myeloma (MM) cells, exhibit expression of certain antigens, against which antibodies delivering cytotoxic effects can be targeted. MM cells express high levels of CD38 glycoprotein. Even though the presence of CD38 is reported on normal cells, its expression is significantly lower than on the MM cells and it consists of a proper therapeutical target. Monoclonal antibodies (mAbs) against CD38 in clinical practice are a successful means of MM treatment [21,56,57,58,59,60,61,62]. Currently, treatment of CD38+ malignancies is possible due to four monoclonal antibodies in clinical trials: daratumumab and isatuximab, which have already received FDA approval, appropriately in 2015 and 2020, MOR202 and TAK079 (Table 1) [21,56,61,62,63].

Interestingly, by pairing two non-competing heavy chain antibodies in a bispecific format, TNB-738 antibody allows for the simultaneous binding of two epitopes on CD38. TNB-738 inhibits CD38’s enzymatic activity, elevating the intracellular NAD+ content and SIRT activity [64]. In January 2022, TNB-738 underwent a single- and multiple-dosing study for targeting CD38 in healthy volunteers (NCT05215912). The aim of the study was to evaluate the safety and tolerability of TNB-738 by the incidence of treatment-emergent adverse events.

The described four typed of mAbs exhibit similar binding affinity and antibody-dependent cell-mediated toxicity (ADCC), however, significant differences can be found in regard to several abilities: to induce complement-mediated cytotoxicity (CDC), to induce antibody-dependent cell-mediated phagocytosis (ADCP), to inhibit enzymatic activities, and to induce direct apoptosis [21,56,67,69]. Furthermore, anti-CD38 mAbs influence the tumor microenvironment by inhibiting suppressive T_reg_ activity and boosting effector T-cell function [21]. Efficacy of the therapy based on usage of each of the four mAbs suggests that ADCC is the main process responsible for defeating MM cells [70]. Interestingly, novel studies in improving the efficacy of isatuximab led to the discovery of SAR442085, a novel anti-CD38 antibody with enhanced ADCC antitumor activity against multiple myeloma [71]. SAR442085 has greater binding affinity than daratumumab and isatuximab for FcγRIIa and FcγRIIIa, which results in NK cells activation against primary plasma cells.

**Figure 3:**
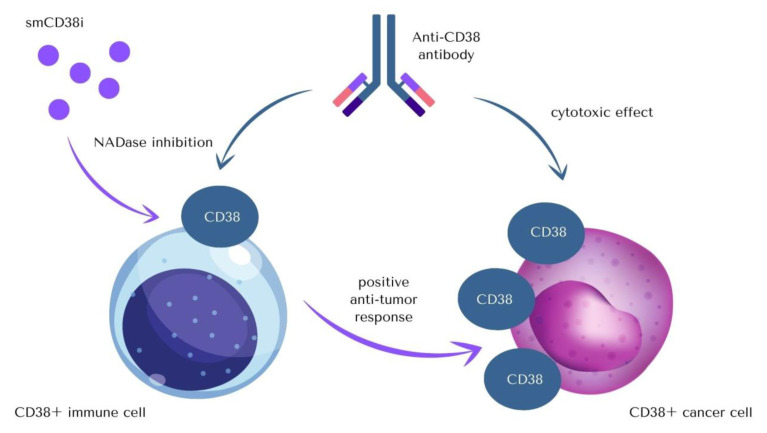
Cytotoxic anti-CD38 antibodies can induce apoptosis of CD38-positive cancer cells through direct or indirect effects. As the level of cellular NAD+ plays a significant role in immune system modulation, small molecule CD38 inhibitors (smCD38i) or monoclonal antibodies that inhibit CD38 activity (CD38imAB) can promote an increase in tissue NAD+ levels and induce positive anti-tumor immune response [18,70,73,74]. There are also several novel anti-CD38 targeting antibodies with the enhanced activity.

NADase activity of CD38 cannot be omitted as there is a possibility that in some parts therapeutical effects of mAbs occur due to inhibition of glycoprotein’s enzymatic activity, which leads to a significant increase of NAD+ level. The impact that anti-CD38 antibodies therapy has on the immune system is thought to be related to a decrease in CD38 enzyme activity [2,72]. Isatuximab is the only mAbs that can inhibit CD38’s NADase activity (Table 1). Modulation of the immune system due to specific regulation of CD38 enzymatic activity might become an important target for age-related diseases therapies aiming to boost intracellular NAD+ level (Figure 3) [29,73,74].

Apart from the therapy based on anti-CD38 antibodies, there are several ways in which the activity of CD38 can be targeted, which creates potential prospect for anticancer—and not only anticancer—therapies, as a multitude of functions performed by CD38 makes it an important part of the pathogenesis of varied conditions.

The variety of inhibitors of CD38 (smCD38i) includes two main functional groups of compounds: covalent inhibitors, which form a bond in the active site at Glu226, and non-covalent inhibitors, which bind to amino acid residues in the active site of the enzyme by weaker interactions [26,75,76,77,78,79,80,81,82]. When it comes to compounds’ chemical structures, there are flavonoids (apigenin, quercetin, and leteolinidin), heterocycles compounds (derivatives of 4-amino-quinoline), and NAD-analogues (carba-NAD and ara-NAD analogs). The therapeutic potential of the described inhibitors is restricted due to their low specificity, as they might influence other NAD-dependent enzymes, not only CD38 [19].

CD38 is one of the potential targets of chimeric antigen receptor (CAR) T cells immunotherapy, in which T lymphocytes are engineered with synthetic chimeric antigen receptors (CAR). The CAR-T cell is therefore an effector T cell that can recognize and eliminate particular cancer cells in a way that is independent of major histocompatibility complex molecules. CAR-T therapy has shown promising activity in hematological malignancies, including lymphoma acute lymphoblastic leukaemia, chronic lymphocytic leukaemia, and multiple myeloma.

CAR-T-38 seems promising in the treatment of B-cell acute lymphoblastic leukaemia (B-ALL) and acute myeloid leukaemia (AML) [83,84]. It is usually used as a ‘last chance’ therapy in patients with poor prognoses. Especially in B-ALL, CAR-T-38 is usually given to patients after failure of other CAR-T therapy, e.g., CAR-T-19, but the safety of this procedure still needs to be evaluated. There was a case of liver and lung damage after the infusion in a patient with relapse and refractory B-ALL because it turned out that CAR-T cells attack also healthy cells such as granulocytes, mononuclears, liver, and lung smooth muscle cells, and pancreatic cells [83]. Moreover, the patient developed a high-grade fever, which, after taking into consideration also her blood tests, indicates the occurrence of CRS [83]. It indicates that more studies are needed to improve this therapy. In 2018 a new clinical trial started. It aims to determine the safety of CAR-T-38 therapy in patients with relapsed B-ALL after CAR-T-19 therapy [85]. The study is not finished yet [85]. Maybe during this trial some problems can be overcome, and it will result in a more optimistic perspective.

However, CAR-T therapy in AML seems even more challenging than in B-ALL because of the absence of a specific target antigen. One of the possibilities is CD38, which is expressed on some AML blast cells. There was a clinical trial with six patients that experienced relapse after allogenic hematopoietic stem cell transplantation [84]. They were given CAR-T-38 infusion and after four weeks four patients achieved remission [84]. There were still adverse effects: five patients presented CRS and one experienced hepatotoxicity, but these disorders did not cause greater problems and all were rather manageable [84]. Now, as with B-ALL therapy, there is a new clinical trial with relapsed AML patients as well [86]. During this study, participants were given CAR-T-38 infusion to confirm the safety of this therapy [86]. Results are not available because the clinical trial is not finished yet [86]. Every new trial is a chance to introduce CAR-T-38 therapy as a safe and effective treatment for patients with poor prognosis.

CD38 may be also a good target for CAR-T in refractory or relapsed multiple myeloma (RRMM). BCMA CAR-T therapy turned out to carry great risk and toxicity and has to be improved (NCT03754764). Bispecific CAR-Ts targeting both BCMA and CD38 were proposed and seem to be a good solution (NCT04351022). Twenty-three patients were given BM38-CAR-T infusion and significant responses were observed. Although CRS and hematological toxicities occurred in almost all patients, they were still manageable, and therapy was considered safe (NCT04351022). The most evident effects were observed in the patients with the extramedullary disease (EMD) that usually has really poor prognosis and bad survival. After the infusion, EMD was eliminated completely or partially, which was seen in CT scans (NCT04351022). In conclusion, BM38-CAR-T therapy seems promising, but more studies are needed to confirm its effectiveness.

## 4. CD38 in Hematological Neoplasms and Diseases

### 4.1. Acute Myeloid Leukemia

CD38 expression is very variable in acute myeloid leukaemia (AML) [87,88]. However, the detection of CD38 can be useful to identify leukemic stem cells (LSC) in AML, especially of the types minimally differentiated (M0) and without maturation (M1), but also in the heterogeneous leukemic cell population of AML CD19(+). It was proved that in LSC CD34(+)CD38(+) the expression of genes related to drug resistance-related ABC transporters, such as *ABCG1*, *ABCB1*, *ABCC1*, *ABCD4*, *ABCB2*, was lower than in LSC CD34(+)CD38(−) [89,90]. The CD34(+)CD38(−) LSC frequency at diagnosis of AML independently predicts shorter overall survival (OS), and CD38 expression can be a positive predictive factor in AML [91]. Corresponding correlation of the enrichment of CD34(+)CD38(−) or CD34(+)CD38(+) cells to higher residual disease may be found after induction treatment [92,93]. Furthermore, CD38 inhibition with anti-CD38 antibody results in high anti-leukemic efficacy in vitro independent of CD38 expression level and induces phagocytosis in AML [94]. It can confirm that CD38 can play the role of a therapeutic target for AML [87].

### 4.2. Acute Lymphoblastic Leukaemia

CD38 expression can be also present in acute lymphoblastic leukaemia (ALL) [95]. The detection of CD38(+)CD58(−) blood cells in B-cell ALL (B-ALL) without Philadelphia chromosome (Ph) can stratify patients to the high-risk group. Such patients had a higher relapse rate and shorter survival. CD38 is an independent adverse prognostic factor in B-ALL Ph(−) [96]. The therapy with anti-CD38 antibody was reported to be effective in the case of CD38(+)Ph(+) recurrent B-ALL [97].

T-cell acute lymphoblastic leukaemia cells have significant CD38 surface expression that remains stable after exposure to multiagent chemotherapy. As a result, CD38 is the following promising target of a novel therapy for paediatric T-ALL patients, too [98].

Due to the unique expression on normal haematogones at multiple stages of normal regeneration of CD38 antigens, it plays a great role in diagnosis of B-ALL and minimal residual disease (MRD) assessment [99].

### 4.3. Chronic Lymphocytic Leukaemia/Small Lymphoma (CLL/SLL), B-Cell Prolymphocytic Leukaemia (B-PLL)

CD38 is expressed to a varying degree on the surface of leukemic cells in CLL/SLL and B-PLL [100]. Nonetheless, the elevated expression (i.e., over 30%) of CD38 in CLL is associated with advanced disease stage, higher incidence of lymphadenopathy, hepatomegaly, high-risk cytogenetics, elevated sβ2m, and sCD23 levels, short lymphocyte doubling time, short time to initiation of the first treatment, poor response to therapy, shorter progression-free survival (PFS) and OS [101,102,103]. CLL cells with unmutated IGHV genes expressed high levels of CD38, however, this association is not absolute and CD38 expression might vary during the disease course [104,105]. High expression of CD105 (endoglin) is predicted to be a potential risk marker, and the therapeutic target in high-risk CLL further correlates with CD38 expression [106].

### 4.4. NK-T Cell Lymphoma

In almost all NK-T cell lymphomas (NKTL), expression of CD38 is detected and may predict poor outcomes [107]. Moreover, Epstein–Barr virus-positive NKTL patients significantly express CD38, with half exhibiting high expression [108]. CD38 interacts with the ligands CD31 and CD31/CD38 and promote the activation and proliferation of different lymphocyte groups. That is why CD38 is a potential therapeutic target in NKTL.

### 4.5. Multiple Myeloma

In multiple myeloma (MM), CD38 antigen is expressed on plasma cells to a higher degree than physiologically [109]. CD38 does not differentiate pathological and physiological plasma cells.

CD38 plays a crucial role in MM thanks to the immunotherapy vastly developing in recent years, which consists of IgG immunoglobulins of anti-CD38 activity [56,110]. It can induce antibody-dependent cellular cytotoxicity, antibody-dependent cellular phagocytosis, complement-dependent cytotoxicity, direct cellular apoptosis, and extracellular ectoenzyme activity modulation [111,112]. Antitumor effects and an immunomodulatory component can be associated with the effect of anti-CD38 immunotherapy action, which leads to depletion of immunosuppressive cells and clonal expansion of cytotoxic T cells [57,68,113,114]. High CD38 expression results in rapid depletion of NK cells after the daratumumab course (anti-CD38 monoclonal antibody), largely eliminating the source of innate immune cells, which could drive even more complete tumor eradication [115,116]. It can be applied as a monotherapy or in combination with proteasome inhibitors (PIs), e.g., bortezomib) with immunomodulatory drugs (IMIDs, e.g., lenalidomide, pomalidomide) [117]. The treatment with anti-CD38 medicaments is still optimizing and being improved in the clinical trials.

The actual registration state of daratumumab in monotherapy in relapsed/refractory MM is daratumumab used in monotherapy (according to the SIRIUS study [118] or in combination with bortezomib (according to the CASTOR study)) or with lenalidomide (according to POLLUX study) [119].

Furthermore, the CASSIOPEIA study proved that the addition of daratumumab to bortezomib, thalidomide, and dexamethasone (VTD) as induction and consolidation improved progression-free survival in patients with autologous stem-cell transplant (ASCT)-eligible newly diagnosed multiple myeloma under 65 years. Moreover, daratumumab maintenance every 8 weeks for 2 years significantly reduced the risk of disease progression or death compared with observation only. Longer follow-up and other ongoing studies will shed further light on the optimal daratumumab-containing post-ASCT maintenance treatment strategy [120]. European Medicines Agency (EMA) approved daratumumab in combination with bortezomib and thalidomide in 2020.

It has been proved that venetoclax (selective BCL-2 inhibitor) in combination with daratumumab and dexamethasone (VenDd) and VenDd with bortezomib contribute to a high rate of deep and durable responses in patients with relapsed/refractory multiple myeloma, especially with t(11;14) [121].

A phase 3 trial of the APOLLO study showed the reduction of risk of disease progression or death using daratumumab plus pomalidomide and dexamethasone compared to therapy with pomalidomide and dexamethasone alone [121].

The ALCYONE trial presented a lower risk of disease progression or death among patients with newly diagnosed multiple myeloma ineligible for stem cell transplantation, after treatment of daratumumab plus bortezomib, melphalan, and prednisone (D-VMP) compared with the same regimen without daratumumab (VMP) [122]. Based on another study, D-VMP prolonged OS in patients with newly diagnosed multiple myeloma ineligible for stem-cell transplantation [123].

The MAIA study demonstrated a significant decrease in risk of disease progression or death among patients with newly diagnosed multiple myeloma who were ineligible for autologous stem-cell transplantation and that received daratumumab plus lenalidomide and dexamethasone, in comparison with patients after lenalidomide and dexamethasone-alone treatment [119,124]. What is more, daratumumab with lenalidomide, bortezomib, and dexamethasone used in induction and consolidation can improve the depth of response in patients with transplant-eligible newly diagnosed multiple myeloma [125].

Daratumumab, originally applied only intravenously, is now accepted to be used subcutaneously due to the COLUMBA study [126]. Finally, in 2021, the EMA approved the usage of daratumumab in combination with bortezomib and dexamethasone.

An alternative treatment to daratumumab, based on the same anti-CD38 activity, is isatuximab, particularly active in patients’ refractory to lenalidomide and PI. This cohort showed PFS prolongation, however, in the HR group the benefit is not so clear [127]. The addition of rituximab to pomalidomide–dexamethasone significantly improves PFS in patients with relapsed and refractory multiple myeloma [128]. Another study established that it is possible to improve PFS in patients with relapsed and refractory multiple myeloma by adding isatuximab to pomalidomide–dexamethasone [128].

**Figure 4:**
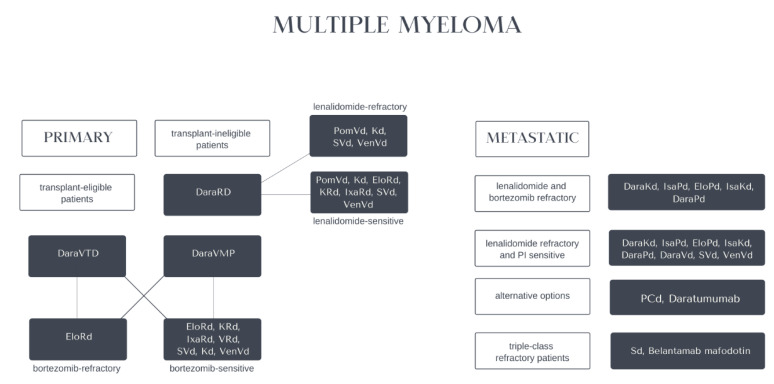
Scheme depicting the therapeutic strategies for patients with primary and metastatic multiple myeloma. Primary neoplasm is divided into transplant eligible and ineligible patients.

Altogether, according to EHA-ESMO Clinical Practice Guidelines for diagnosis, treatment and follow-up in multiple myeloma, recommended use of anti-CD38 antibodies (including daratumumab and isatuximab) is wide, i.e., DaraRd (daratumumab, lenalidomide, dexamethasone) or DaraVMP (daratumumab, bortezomib, melphalan, prednisone) as the first-option treatment of transplant-ineligible patients, DaraVTD (daratumumab, bortezomib, thalidomide, dexamethasone) as the first-option induction of transplant-eligible patients; in the second-line after VRd (bortezomib, lenalidomide, dexamethasone): DaraRd (daratumumab, lenalidomide, dexamethasone), DaraKd (daratumumab, carfilzomib, dexamethasone), IsaKd (isatuximab, carfilzomib, dexamethasone) for lenalidomide-sensitive MM, DaraKd (daratumumab, carfilzomib, dexamethasone) for lenalidomide-refractory MM, DaraRd (daratumumab, lenalidomide, dexamethasone), DaraKd (daratumumab, carfilzomib, dexamethasone), DaraVd (daratumumab, bortezomib, dexamethasone), IsaKd (isatuximab, carfilzomib, dexamethasone) for bortezomib-sensitive MM, DaraKd (daratumumab, carfilzomib, dexamethasone) for lenalidomide and bortezomib-refractory MM and at second or subsequent relapse: DaraKd (daratumumab, carfilzomib, dexamethasone), DaraPd (daratumumab, pomalidomide, dexamethasone) for lenalidomide and bortezomib refractory MM; DaraKd (daratumumab, carfilzomib, dexamethasone), IsaPd (isatuximab, pomalidomide, dexamethasone), IsaKd (isatuximab, carfilzomib, dexamethasone), DaraPd (daratumumab, pomalidomide, dexamethasone), DaraVd (daratumumab, bortezomib, dexamethasone) for lenalidomide refractory and proteasome inhibitor sensitive MM; alternatively, daratumumab as a monotherapy [74]. Inherited genetic variation rs6449182 in the CD38 gene may impact the risk of MM development [129]. Novel therapeutic options for multiple myeloma are presented in Figure 4.

In 2022, the Food and Drug Administration (FDA) and Committee for Medicinal Products for Human Use (CHMP) of the European Medicines Agency (EMA) approved the use of Ciltacabtagene Autoleucel (CARVYKTI^®^) against multiple myeloma cells. The biological drug is a CAR-T cell, composed of B-cell maturation antigen (BCMA)-targeting single-domain antibodies. The drug may be used for adults with relapsed and refractory multiple myeloma. Besides, the patients have to receive an immunomodulatory agent, a proteasome inhibitor, and an anti-CD38 antibody before the use of CAR-T cell therapy.

### 4.6. Aplastic Anaemia

T cells from aplastic anaemia (AA) patients, especially the CD38+CD8+ T cell subset, are increased in AA patients with CD38+CD8+ T. They have higher pro-inflammatory and proliferative capacity that may contribute to the pathologic progression in AA. CD38+CD8+ T cells infiltrate the bone marrow (BM) of AA patients and may destroy BM. Such cells exhibit higher potential for survival and proliferation and promote inflammation. Complement also shows enrichment in CD38+CD8+ T cells suggests the involvement of the complement system [130,131].

CD38+CD8+ T cells characterize by the expression of genes involved in homeostasis in the condition of hypoxia. Due to the possible function in maintaining homeostasis in hypoxia, CD38 may play an important role in the activation of CD8+ T cells in the BM of AA patients [132].

### 4.7. Immune Thrombocytopenic Purpura

In immune thrombocytopenic purpura (ITP), the expression of CD38 and CD56 is significantly lower before treatment than after it. CD38 expression is also strongly correlated with platelet count before and after treatment. The mechanism of this correlation is associated with the regulatory B-cells (Breg) subset characterized by CD19+CD38hiCD24hi expression. The cells promote peripheral tolerance and diminish the function of autoreactive T-helper CD4+ cells via the production of interleukin (IL) 10. CD38-positive Breg cells are responsible for the maintenance of peripheral tolerance. Their reduced numbers negatively correlate with the increase in autoreactive B- and T cells as well as disease activity and the onset of ITP. A low expression of CD38 cells, as well as the positive correlation of this marker with platelet counts, may have independent prognostic value in ITP patients, which may be related to the loss of peripheral tolerance [133].

### 4.8. Immunoglobulin Light-Chain (AL) Amyloidosis

The pathomechanism of immunoglobulin light-chain (AL) amyloidosis consists of clonal expansion of CD38+ plasma cells that produce misfolded immunoglobulin light chains, which form amyloid fibrils deposited in tissues [134]. The addition of daratumumab to the treatment of newly diagnosed AL amyloidosis with bortezomib, cyclophosphamide, and dexamethasone could improve hematologic complete response and survival free from major organ deterioration or hematologic progression [76]. According to the results of the ANDROMEDA study, daratumumab received subcutaneously in combination with cyclophosphamide, bortezomib, and dexamethasone (VCd) was mostly well tolerated by patients with newly diagnosed AL amyloidosis and demonstrated robust hematologic and organ responses in these patients [135]. The data presented by Dispenzieri et al. proved that ixazomib with dexamethasone may be a potentially beneficial treatment option for patients with relapsed/refractory AL amyloidosis after 1–2 prior lines [136]. Daratumumab s.c. was registered in combination with VCd in the treatment of AL amyloidosis.

### 4.9. Other Hematological Abnormalities

In Burkitt lymphoma, CD38 antigen can be detected in combination with LIM Domain Only 2 (LMO2) (−). CD38-positive staining was also observed in a case of unusual B-CLL presentation due to the complication of Burkitt transformation or Burkitt-like high-grade transformation at initial presentation under the wide definition of Richter syndrome, described by Chatzidimitriou et al. [137,138].

Immune anomalies in Evans syndrome CD38 can involve a broad spectrum of T-cell dysregulation [139]. The presence of dysplastic myeloblasts with the absence of CD38 antigen strongly suggests myelodysplastic syndrome [140].

A threshold of >7% CD38high/HLA-DR+ cells among CD8+ T cells has strong positive and negative predictive values for distinguishing haemophagocytic lymphohistiocytosis from early sepsis or healthy controls [141]. Also, the CD38 expression level corresponds with the level of the aggressive clinical course of hairy cell leukaemia [142].

During the pathology diagnostic process, CD38 antigen can also be found as a marker of differentiation, especially into plasma cells, in malignancies, such as lymphoplasmacytic lymphoma (Waldenström macroglobulinemia), MYC translocated aggressive B-cell lymphomas, ALK-positive large B-cell lymphoma, high-grade B-cell lymphoma (HGBL; including HGBL with rearrangement of *MYC*, *BCL2*, *BCL6* genes), plasmablastic lymphoma, plasmablastic plasma cell myeloma, anaplastic large cell lymphoma, and primary effusion diffused large B-cell lymphoma [143,144,145,146,147]. The CD38 expression may vary in diffuse large B-cell lymphoma (DLBCL) associated with chronic inflammation, lymphomatoid granulomatosis, and HHV-8-positive DLBCL (Table 2).

## 5. CD38 Outside Hematology

### 5.1. Non-Hematological Malignancies

CD38 could play a carcinogenic role in nasopharyngeal cancer (NPC), by inhibiting cell aging, promoting cell proliferation, cell metastasis, and the S phase induction. Furthermore, CD38 increases the concentration of ATP, lactic acid, cAMP, and human ADP/acrp30 concentration in NPC cells. It regulates the metabolism-regulating signaling pathways associated with tumor protein 53 (TP53), hypoxia-inducible factor-1α, and sirtuin 1, too [148].

CD38+ tumor-infiltrating immune cells ((TIICs), including B-cells and myeloid cells) density increase the following progression to castration-resistant prostate cancer (CRPC) and shortens OS [149]. In metastatic CRPC, CD38 mRNA expression correlates with upregulated immune signaling pathways, e.g., with the excretion of IL-12, IL-23, and IL-27, immunosuppressive adenosine signaling and T cell exhaustion signatures. CD38 expression in CRPC cells lowers intracellular NAD+. It results in cell cycle arrest and expression of Cyclin-Dependent Kinase Inhibitor 1A (p21Cip1/CDKNA1) [150]. Parallelly, CD38 diminishes glycolytic and mitochondrial metabolism, activating AMP-activated protein kinase and inhibiting fatty acid and lipid synthesis. Modulation of NAD+ by CD38 induces differential expression of the transcriptome, producing a gene expression signature indicative of a non-proliferative phenotype [151]. CD38 is silenced in tumor cells likely because metabolically active cells rely upon NAD+ and NAD+-dependent enzymes for glycolysis and mitochondrial biogenesis. In leucocytes, upregulation of CD38 inhibits effector function since NAD+-dependent signaling, potentially through the NAD+-SIRT1-FOXO1 axis, promotes effector T cell differentiation and antitumor potential [29,72]. It is possible that CD38 blockade increases intracellular NAD+ level and activates T cell-mediated immunity, despite the presence of an immunosuppressive. In localized and metastatic prostate cancer, CD38 epigenetic regulation was found. It regulates extracellular NAD+ in epithelial cells [72].

A significant expansion of CD38+ monocytic and polymorphonuclear myeloid-derived suppressor cells (M-MDSCs and PMN-MDSCs) with a tendency to increase CD38 expression on M- and PMN-MDSCs can be observed in peripheral blood mononuclear cells of patients with colorectal cancer (CRC). The CD38+ M-MDSCs are found to be immunosuppressive in comparison with mature monocytes. CD38+ M- and PMN-MDSC are present significantly more frequently in CRC patients previously treated when compared with treatment-naive patients [152].

In glioma cells, CD38 has an impact on the intracellular ATP levels and the survival of C6 glioma cells, which makes CD38 a potential therapeutic target for gliomas [153].

### 5.2. HIV Infection, AIDS

CD38 NADase activity is increased with human immunodeficiency virus (HIV) infections in vitro, reducing levels of NAD in leukocytes, including chronically over-activated T lymphocytes [154]. An increase in CD38+ immune cells is reported in HIV infections, likewise in infections with Epstein–Barr virus and cytomegalovirus [155,156]. CD38 also indirectly reflects the viral load. Anti-retroviral therapy significantly reduces the proportion of CD38-positive lymphocytes in HIV-infected patients. Viral load in the body is an indicator of the antiviral effect of the treatment that is strictly associated with the CD38-positive lymphocytes count [157]. Therefore, CD38 expression can sensitize cells to HIV infection and enhance HIV replication.

Moreover, the primary effusion lymphoma usually coexisting with CD38 expression and HIV infection can be effectively treated with daratumumab, with a clinical response confirmed by imaging and a reduction in the viral load [158]. Though, as this lymphoma is very rare, large cohort clinical trials are difficult to be held.

### 5.3. COVID-19

CD38-mediated thrombosis may be observed in COVID-19 disease. What is more, CD38 is needed to mediate NAD+-dependent bacterial engulfment but also to rearrange the cytoskeleton in phagocytes and to perform adenosine diphosphate ribose-dependent signaling required for the migration of immune cells to the site of infection [159,160]. As a result, CD38 may play a substantial role in enhancing symptoms of SARS-CoV-2 infection and make the risk of secondary bacterial infection higher [38,161].

CD38 activation and NAD+ decline can be recognized as features of aging and thus may be seen as modulators of COVID-19 disease in the elderly. Hyperinflammation in COVID-19 may lead to CD38 activation and NAD+ degradation, predisposing to severe outcomes, including tissue fibrosis and tissue damage, particularly in the elderly.

CD38-targeted therapies may predispose though to opportunistic bacterial infections, especially in primary viral respiratory tract infections. It was confirmed by the observation taken over cancer patients treated with daratumumab [162].

However, in the time of COVID-19, in the treatment of MM, a DaraRD (full doses of daratumumab every 4 weeks, lenalidomide, dexamethasone 40 mg p.o. firstly with de-escalation when no infusion reaction documented) 28-day cycle is recommended both for standard-risk patients as well as for high-risk patients. In standard-risk patients, after 10–12 cycles, switching to maintenance therapy with lenalidomide can be considered, when the goals of therapy are achieved [163]. In relapsed MM with no high-risk genetic features and without daratumumab as part of the induction, daratumumab-based regimens are recommended. De-intensified regimens for daratumumab should be used based on patient risk and response [164]. Nevertheless, due to the COVID-19 pandemic, a DaraVTD regimen is one of the preferred induction therapies.

When it comes to the anti-SARS-CoV-2 vaccines, an ongoing treatment without daratumumab is associated with a higher likelihood of response for BNT162b2 vaccine in MM patients [165]. At once, daratumumab with lenalidomide leads to a significantly lower response rate as anti-CD38 antibodies targets CD38 on all plasma cells, i.e., antibody producer cells. Though, the evidence about immunogenicity after daratumumab is not clear [166]. Regardless, checking levels of anti-SARS-CoV-2 neutralizing antibodies may be clinically useful in identifying patients’ responses due to the possible increased risk of SARS-CoV-2 infection and possible benefits from a booster vaccine or prophylactic treatment [167].

### 5.4. Gastrointestinal Diseases

CD38 is expressed to a higher degree in patients with Crohn’s disease and patients with ulcerative colitis. The expression is higher in inflamed tissues than in non-inflamed tissues. CD38 is located in F4/80-positive cells.

In celiac diseases, CD38 expression on gluten-specific T cells augments remarkably. A single gluten challenge can induce a response of gluten-specific T cells and stays not inferior to the three-day challenge regarding CD38 expression. It suggests CD38 expression as an alternative to outcome measures concerning celiac disease patients [168]. Although the expression of CD38 may be a possible target for the therapy of gastrointestinal diseases, there are currently no data on the use of anti-CD38 antibodies in the therapy.

### 5.5. Pulmonary Injury Induced by Escherichia Coli

CD38 deficiency increases the expressions of pro-inflammatory factors, IL-1β, and chemokine MCP-1, and aggravates pulmonary injury by TLR4/ERK/NF-kB signaling pathways [169]. CD38 could activate MAPK/NF-kB signaling pathway in mice sepsis caused by pulmonary injury induced by E. coli, leading to the release of inflammatory cytokines, such as IL-1b, and inflammasome NLRP3 [170]. Aggravation of pulmonary injuries and inflammatory reactions is caused by the up-regulation of ERK1/2 and NF-kB pathways due to the CD38 deficiency. The expressions of phosphorylated ERK1/2 and NF-kB/p65 then raise significantly. The phosphorylation of ERK1/2 and NF-kB/p65 yield increase in CD38 ^−^/^−^ + E. coli group through TLR4. ERK1/2 and NF-kB/p65 could be then highly activated and play a crucial function in the inflammatory response in sepsis in CD38 deficiency [171]. The activation of ERK1/2 and NF-kB p65 results in exacerbation of pulmonary injury and inflammatory response in CD38 ^−^/^−^ sepsis mice.

### 5.6. Neurological Diseases

CD38 controls brain NAD bioavailability and the activity of NAD-dependent enzymes crucial for neuronal survival. Therefore, inhibition of CD38 enzymatic activity leading to increased NAD levels might be a neurodegenerative disease treatment target [172]. The immunosuppressive effect of anti-CD38 antibodies on plasma cells and plasmablasts could be also useful against autoimmune neurological disorders such as multiple sclerosis [173]. It was confirmed by Roboon et al., who discovered the link between CD38-mediated neuroinflammation and NAD+ consumption [174]. According to them, boosting NAD+ by CD38 inhibition and nicotinamide riboside supplementation directly suppresses neuroinflammation in the brain. CD19+CD24hiCD38hi transitional B-cells may be a potential biomarker for disease activity [175]. Activated memory B-cells with intermediate and high expression of CD38 are susceptible to fingolimod, so their decrease is observed after the course of the treatment of multiple sclerosis [176,177]. Furthermore, after fingolimod application, the proportion of regulatory B-cells (CD38+CD27−CD24+CD5+) is significantly increased as compared to treatment-naïve multiple sclerosis patients and healthy controls, and significantly more of them produce IL-10 [178]. When it comes to IFN-β treatment, it increases the absolute number of regulatory CD19+CD24++CD38++ transitional B-cells and CD8+CD38+ T lymphocytes in peripheral blood as compared to treatment-naïve and Copaxone-treated patients [179,180]. Finally, circulating CD24hiCD38hi B-cells increase in percentage in the majority of patients with relapsed or refractory multiple sclerosis (RRMS) after 4–6 months and 12 months of treatment with BG-12 (dimethyl fumarate) [181].

Increased expression of CD38 can be observed during the kindling procedure in the hippocampus, which suggests it as one of the most important NAD+ degrading enzymes during epilepsy progression. Moreover, the expression level of the gene-encoding mGluR1 (one of CD38 metabolite related proteins) significantly changes then. It means that changes in the expression of the CD38/cADPR signaling pathway may be crucial in epileptogenesis [182].

### 5.7. Systemic Lupus Erythematosus

CD38 can undermine the cytotoxic function of CD8+ T lymphocytes in systemic lupus erythematosus (SLE). This fact can be associated with the increased susceptibility of SLE patients to infections [55]. They also present higher frequencies of activated CD38+HLA-DR+ T cells than healthy people, especially patients with the low disease when it comes to circulating activated CD38+HLA-DR+ CD4+ and CD8+ T cells. Presenting with activated T cells and a hyperactive metabolic signature may allow for correcting aberrant immune activation through targeted metabolic inhibitors [183]. Ostendorf et al. reported clinical and serologic responses in two patients treated with daratumumab who had suffered from treatment-refractory multisystem lupus before [184]. The observations confirmed a significant depletion of long-lived plasma cells, reduction of interferon type I activity, as well as downregulation of T-cell transcripts associated with chronic inflammation. Moreover, laboratory research confirms enhanced degranulation of both healthy and SLE NK cells, and as a result killing circulating plasma cells, in response to daratumumab [185,186].

### 5.8. Transplant Rejection

The study of Liu et al. suggested circulating CD19(+)CD86(+)CD38(+) B lymphocytes may be one of the biomarkers in renal transplant recipients with antibody-mediated acute rejection. Such B-cells can promote the differentiation of B-cells into plasma cells by activating B-cells, thereby promoting disease progression [187].

Daratumumab may be applied to reduce donor-specific antibodies in case of antibody-mediated rejection (AMR) in lung transplantation, inducing plasma cell death through multiple mechanisms including complement-dependent cytotoxicity, antibody-dependent phagocytosis and apoptosis [188].

Corresponding targeting of CD38 with daratumumab for plasma cell and NK cell depletion may be also useful in the treatment of chronic active antibody-mediated kidney allograft rejection (including ABO-incompatible), as well as AMR after heart transplantation [189,190,191,192,193].

### 5.9. Arthritis

CD38 could regulate collagen-induced arthritis through the NF-κB pathway. As a consequence, it could be a novel target for the treatment of autoimmune inflammatory joint disease, including rheumatoid arthritis [185,194]. What is more, treatment of cynomolgus monkeys suffering from collagen-related arthritis with TAK-079 was well tolerated and reduced disease progression and symptoms. It was confirmed with arthritis scores, inflammatory parameters, a decrease in symptoms, as well as histopathology, morphometry and radiology [195]. Table 3 summarizes the role of CD38 in non-hematological diseases.

## 6. Experimental Medicine Involving CD38—Clinical Trials

### 6.1. Daratumumab and Isatuximab in Multiple Myeloma, NK- and T-Cell Lymphomas, and B-Cell Malignancies

According to ClinicalTrials.gov, there are currently 156 studies conducted across the world investigating the safety and efficiency of daratumumab alone or in combination with different drug regimens in the treatment of various kinds of multiple myeloma. Further, 43 trials investigate the effectiveness of isatuximab in the treatment of MM in different therapeutic strategies. Even though both of these mAbs were FDA-approved in 2015 [118] and 2020, respectively [65], much research still has to be done in this field.

The main objective of the NCT01084252 randomized clinical trial was to evaluate SAR650984 (isatuximab) as a single agent or in combination with dexamethasone in patients suffering from hematological malignancies, including relapsed/refractory multiple myeloma (RRMM). As a single agent, in patients with RRMM, isatuximab was found to be most effective at doses > 10 mg/kg [196]. In patients with RRMM, who underwent on average four prior therapies, adding dexamethasone increased response rates from 23.9% to 43.6% and survival outcomes, with no debilitating effects on patients’ safety [109].

NCT03275285, a phase 3 trial, aimed to prove the superiority of treatment of patients with RRMM with isatuximab in combination with carfilzomib and dexamethasone in comparison with carfilzomib and dexamethasone alone [197]. Patients with multiple myeloma often develop renal impairment (RI) [198]. In this trial, the addition of isatuximab to therapy improved clinical outcomes in patients with renal impairment [197].

The main objective of the NCT01749969 study is to assess the best dosage and scheduling of isatuximab given with lenalidomide and dexamethasone in patients with RRMM [62]. This trial found out that a specific genetic makeup controlling NK cell function (precisely KIR3DL2+ and HLA-A3/11+ with FCGR3A-158V markers) causes greater NK-mediated isatuximab-dependent cytolysis against multiple myeloma cancer cells, which results in higher PFS for those patients. Lacking these components results in decreased efficiency of the treatment and detecting them beforehand (also identifying the presence of KIR2DL1+ and HLA-C2C2+, which may cause a lower response to the drugs) may be beneficial for deciding on the treatment [199].

The main objective of NCT04751877 ongoing, recruiting trial is to establish the role of proteasome inhibitors (bortezomib) in treatment for non-transplant-eligible non-frail newly diagnosed multiple myeloma. Patients enrolled in this trial are above 65 and under 80 years old. The treatments used include isatuximab + lenalidomide + dexamethasone with or without bortezomib (NCT04751877).

NCT00574288 (GEN501) study established daratumumab as a safe single agent in treatment of RRMM [56]. It also found (along with NCT01985126 (SIRIUS) trial) that resistance to daratumumab is associated with increased expression of complement-inhibitory proteins (CIP) and a response to such therapy is directly linked with CD38 expression levels on cancer cells. Taken together, a therapeutic approach that targets CD38 and CIP expression levels might be beneficial when treating patients suffering from multiple myeloma [60].

In the past couple of decades, multiple different treatment regimens for treating multiple myeloma have been developed [200]. Adding the use of anti-CD38 antibodies can be considered a breakthrough in managing this disease [21,125]. The purpose of the NCT01998971 study is to evaluate tolerability, safety, and the most efficient dose of daratumumab in combination with different treatment regimens for MM, including bortezomib–dexamethasone, bortezomib–melphalan–prednisone, bortezomib–thalidomide–dexamethasone, pomalidomide–dexamethasone, carfilzomib–dexamethasone, and carfilzomib–lenalidomide–dexamethasone. Although the study is not yet finished, it has some interesting findings. Daratumumab in combination with pomalidomide and dexamethasone-induced a deep, fast, and long-lasting response in patients with RRMM with no new adverse effects, compared to single-agent daratumumab, except for lower neutrophil count [127]. Daratumumab with carfilzomib and dexamethasone showed similar results [201], while also demonstrating the possibility of splitting the first dose of daratumumab [201,202].

When compared with the NCT03158688 study, where carfilzomib was administered twice a week at 56 mg/m^2^ (KdD56) to once a week at 70 mg/m^2^ (KdD70), KdD70 once a week is comparable to twice a week KdD56 regarding efficacy and safety, while at the same time being a more convenient option [203].

The NCT02195479 study compared the safety and efficiency of bortezomib–melphalan–prednisone treatment with or without daratumumab in patients with NDMM ineligible for high-dose chemotherapy and autologous stem cell transplant. Adding daratumumab to the regime resulted in a lower risk of disease progression or death [122]. It is generally more beneficial to include this anti-CD38 mAb in the treatment of NTE NDMM in terms of overall survival and progression-free survival.

When taken together with the NCT02252172 study, (lenalidomide + dexamethasone with or without daratumumab in patients with similar eligibility criteria) we can conclude that durable minimal residual disease (MRD) negativity is correlated with longer progression-free survival (PFS). Besides, therapies that include daratumumab are associated with both higher levels of MRD negativity and its higher durability.

A robust analysis by Cavo et al., of four phases 3 studies (NCT02195479, NCT02252172, NCT02136134, NCT02076009) has shown that a complete response or better (≥CR) with MRD-negative is a significant prognostic factor for PFS in TIE NDMM and RRMM. All the data gathering the current clinical trials are summarized in Table 4.

### 6.2. Daratumumab and Isatuximab in Lymphomas and Acute Lymphoblastic Leukemias

There are eight ongoing trials investigating the safety and efficiency of daratumumab in patients suffering from different types of HL, non-HL, and ALLs and two active trials investigating isatuximab in different types of HL, non-HL, and ALLs.

The purpose of the NCT04763616 ongoing, recruiting study is to determine the efficacy of cemiplimab and isatuximab in relapsed or refractory NK or T-cell lymphoid malignancy. Cemiplimab is a monoclonal antibody that binds to the programmed cell death receptor 1 (PD-1). It is approved for use in cutaneous squamous cell carcinomas. Both Extranodal NK/T cell lymphoma and aggressive NK cell leukaemia cancer cells express PD-L1, hence the use of a PD-1 inhibitor might be beneficial. Additionally, using an anti-CD38 antibody (Isatuximab) that mediates not only antibody-related cytotoxicity but also direct anti-tumor effect might be advantageous in the treatment of these diseases.

The primary objective of the NCT02999633 study was to evaluate the efficacy of isatuximab in the treatment of T-ALL or T-LBL. Unfortunately, most of the patients enrolled (11) had a progression in the disease and 10 patients had treatment-related emergent adverse events, so the trial was terminated. Eleven patients died during the treatment, and five died within a month from the last dose due to treatment-related effects. Nevertheless, CD38 is highly expressed on T-ALL blasts, and preclinical studies have proven that isatuximab shows significant ADCP and ADCC activity against acute lymphoblastic leukaemia cell lines (both T-ALL and B-ALL), due to the high CD38 expression mentioned earlier, so it is likely that a therapeutic approach using anti-CD38 methods in the treatment of T-ALL, T-LBL or B-ALL will be established in the future. Data concerning the subject are summarized in Table 5.

### 6.3. Daratumumab and Isatuximab in AML

Three clinical trials evaluate the use of daratumumab in AML: NCT03067571, NCT03537599, and NCT04614636. The others are summarized in Table 6.

As we can see, there are multiple experiments carried out across the world analyzing the effectiveness of this two anti-CD38 mAbs on numerous types of hematological malignancies. The findings seem to be promising, and FDA approval for the use of these mAbs in the treatment of other types of cancers might await in the future.

## 7. Conclusions

CD38 plays an important role in cancer and non-cancerous diseases. By influencing the NAD+/cADPR metabolism and the activity of T cells, CD38 allows for mitigating the immune response in autoimmune diseases. Moreover, targeting CD38 on AML blast cells leads to the induction of cell death. Antibody-dependent cell cytotoxicity occurs after targeting the neoplasms with anti-CD38 antibodies, such as isatuximab or daratumumab. The efficacy of anti-CD38 therapy was proved against multiple myeloma, NK cell lymphomas, and CD19- B-cell malignancies in various clinical trials and laboratory experiments.

## Figures and Tables

**Table 1:** Types of anti-CD38 antibodies used in the therapy of CD38+ malignancies and mechanisms of their action.

Name of an Antibody	Mechanism of Action	References
Isatuximab	Allosteric inhibition of NADase activity	[14,21,62,65,66,67]
Daratumumab	Cytotoxic effect or clearance of CD38+ cells	[56,57,68]
TAK-079 (Takeda)	Cytotoxic effect or clearance of CD38+ cells	[21,63]
MOR-202 (Morphosys)	Cytotoxic effect or clearance of CD38+ cells	[21,69]

**Table 2:** Hematological diseases involving the role of CD38 in the therapy.

Affliction	Role of CD38	Progression in Treatment	References
Acute myeloid leukaemia	Antiproliferative effect, autophagy induction	Several effective therapy attempts	[87,88]
Acute lymphoblastic leukaemia	Independent adverse prognostic factor in B-ALL Ph(−)	The promising target of a novel therapy for paediatric T-ALL patients	[97]
Chronic lymphoblastic leukaemia (CLL), Small lymphocytic lymphoma (SLL), B-cell prolymphocytic leukaemia (B-PLL)	Associated with advanced disease stage, higher incidence of lymphadenopathy, hepatomegaly, high-risk cytogenetics, short lymphocyte doubling time, short time to initiation of the first treatment, poor response to therapy, shorter progression-free survival	Prognostic marker–presence correlates with the worse course	[100]
NK-T cell lymphoma	Poor prognostic marker, CD31/CD38, promotes the activation and proliferation of different lymphocyte groups	Therapeutic target	[107]
Multiple myeloma	Expressed on plasma cells to a higher degree than physiologically	Anti-CD38 immunotherapy action, which leads to depletion of immunosuppressive cells and clonal expansion of cytotoxic T cells	[126]
Aplastic anaemia	Higher proinflammatory and proliferative capacity	Prognostic marker	[132]
Immune thrombocytopenic purpura	CD38+ Breg cells induce peripheral tolerance and diminish the function of autoreactive T-helper CD4+ cells via the production of IL-10	Prognostic marker presence correlates with the worse course	[133]
Immunoglobin light-chain amyloidosis	Clonal expansion of CD38+ plasma cells that produce misfolded immunoglobulin light chains	Daratumumab registered in combination with VCd in the treatment of AL amyloidosis	[136]
Burkitt lymphoma	CD38+ cells regulate the potential of CD8+ cells involved in the pathogenesis of the disease	Prognostic marker	[137,138,140,141,142,143,144,145,146,147]
Evans syndrome			
Lymphoplasmacytic lymphoma			
MYC translocated aggressive B-cells lymphoma			
ALK-positive large B-cells lymphoma			
High-grade B-cell lymphoma (HGBL)			
Plasmablastic lymphoma			
Plasmablastic plasma cell myeloma			
Anaplastic large cell lymphoma			
Primary effusion diffused large B-cell lymphoma			

**Table 3:** Role of CD38 in non-hematological diseases.

Affliction	Role of CD38	References
Non-hematological malignancies	Nasopharyngeal cancer (NPC): cell aging inhibition, cell proliferation promotion, cell metastasis, and conversion to the S phase; increase in the concentration of ATP, lactic acid, cAMP, and human ADP/acrp30 concentration in NPC cells, regulation of the metabolic associated signaling pathways associated with tumor protein 53 (TP53), hypoxia-inducible factor 1α (HIF-1α) and sirtuin 1 prostate cancer (PC): progression to castration-resistant PC, OS shortening, upregulation of excretion of IL-12, IL-23, and IL-27, immunosuppressive adenosine signaling and T cell exhaustion signatures, decrease in glycolytic and mitochondrial metabolism, activating AMP-activated protein kinase and inhibiting fatty acid and lipid synthesis colorectal cancer: expansion of CD38+ monocytic and polymorphonuclear myeloid-derived suppressor cells (M-MDSCs and PMN-MDSCs) with a tendency to increase CD38 expression on M- and PMN-MDSCs glioma: impact on the intracellular ATP levels and the survival of C6 glioma cells	[29,72,148,149,151,169,170]
AIDS	Sensitizing cells to HIV infection and enhancing HIV replication	[150]
COVID-19	CD38-mediated thrombosis, enhancing symptoms of SARS-CoV-2 infection	[159,160]
Gastrointestinal diseases	Expression is visibly higher in patients with ulcerative colitis and Crohn’s disease	[168]
Pulmonary injury induced by *Escherichia coli*	Activation of MAPK/NF-kB signaling pathway	[170]
Neurological diseases	Controlling NAD bioavailability in the brain; expression crucial in epileptogenesis	[174]
Systemic lupus erythematosus	Undermining cytotoxic function of CD8+ T lymphocytes	[185,186]
Transplant rejection	Biomarker in renal transplant recipients with antibody-mediated acute rejection; promotion of disease progression	[188]
Collagen-induced arthritis	Regulation of collagen-induced arthritis	[185,194]

**Table 4:** Daratumumab and Isatuximab in Multiple Myeloma, NK- and T-cell lymphomas, and B-cell malignancies.

Identifier	Patients Number	Recruitment Status	Condition or Disease	Target Antigen	Therapy Protocol	Short Description
NCT01084252	351	Active, not recruiting	Hematological Malignancy	CD38	Drug: Isatuximab SAR650984 Drug: Dexamethasone	Multiple Intravenous Administrations of a Humanized Monoclonal Antibody (SAR650984) Against CD38 in Patients with Selected CD38+ Hematological Malignancies
NCT02332850	89	Recruiting	Multiple Myeloma	CD38	Biological: Isatuximab Drug: Carfilzomib Drug: Dexamethasone	SAR650984 in Combination with Carfilzomib for Treatment of Relapsed or Refractory Multiple Myeloma
NCT01749969	60	Active, not recruiting	Plasma Cell Myeloma	CD38	Drug: isatuximab SAR650984, lenalidomide and dexamethasone	SAR650984 (Isatuximab), Lenalidomide, and Dexamethasone in Combination in RRMM Patients
NCT04763616	37	Recruiting	Natural Killer/T-cell Lymphoma, Relapsed Natural Killer/T-cell Lymphoma, Refractory Natural Killer/T-cell Lymphoma	CD38	Drug: Isatuximab Drug: Cemiplimab	Isatuximab and Cemiplimab in Relapsed or Refractory Natural Killer/T-cell Lymphoid Malignancy (ICING)
NCT04751877	270	Recruiting	Multiple Myeloma, Myeloma	CD38	Drug: Isatuximab Drug: Lenalidomide Drug: Bortezomib Drug: Dexamethasone	Isatuximab + Lenalidomide + Dexamethasone With/Without Bortezomib in de Novo Non-Frail NTE Multiple Myeloma Elderly Patients
NCT02513186	90	Active, not recruiting	Plasma Cell Myeloma	CD38	Drug: isatuximab SAR650984 Drug: lenalidomide Drug: bortezomib Drug: cyclophosphamide Drug: dexamethasone	Isatuximab in combination with CyBorD in Newly Diagnosed Multiple Myeloma (MM)
NCT00574288	104	Completed	Multiple Myeloma	CD38	Drug: Part 1: Daratumumab Drug: Part 2: Daratumumab Other: Methylprednisolone Other: Dexamethasone	Daratumumab Safety Study in Multiple Myeloma
NCT01998971	242	Active, not recruiting	Multiple Myeloma	CD38	Drugs: Daratumumab, Velcade, Pomalidomide, and others	JNJ-54767414 (HuMax CD38) (Anti-CD38 Monoclonal Antibody) in Combination with Backbone Treatments for the Treatment of Patients with Multiple Myeloma
NCT02195479	706	Active, not recruiting	Multiple Myeloma	CD38	Drug: Velcade Drug: Melphalan Drug: Prednisone Drug: Daratumumab IV Drug: Dexamethasone Drug: Daratumumab SC	Combination of Daratumumab and Velcade (Bortezomib) Melphalan-Prednisone (DVMP) Compared to Velcade Melphalan-Prednisone (VMP) in Participants with Previously Untreated Multiple Myeloma
NCT03236428	42	Active, not recruiting	Monoclonal Gammopathy, Smoldering Multiple Myeloma	CD38	Drug: Daratumumab	CD38 Antibody Daratumumab in Patients with High-Risk MGUS and Low-Risk Smoldering Multiple Myeloma
NCT05182073	168	Recruiting	Multiple Myeloma, Myeloma	CD38	Drug: FT576 Drug: Cyclophosphamide Drug: Fludarabine Drug: Daratumumab	FT576 as Monotherapy and in Combination with Daratumumab in Subjects with Relapsed/Refractory Multiple Myeloma
NCT04430530	100	Recruiting	CD19 Negative B-cell Malignancies	CD38, CD22, CD123, CD10, CD20	Biological: Infusion of 4SCAR-T specific to CD22/CD123/CD38/CD10/CD20	4SCAR-T Therapy Post CD19-targeted Immunotherapy
NCT03767751	80	Recruiting	Multiple Myeloma	CD38, BCMA	Biological: Dual Specificity CD38 and BCMA CAR-T Cells	Dual Specificity CD38 and BCMA CAR-T Cell Immunotherapy for Relapsed or Refractory Multiple Myeloma
NCT03754764	80	Recruiting	Relapsed B-cell Acute Lymphoblastic Leukaemia After CD19 CAR-T ACI	CD38	Biological: Specificity CD38 CAR-T Cells	CD38 CAR-T Cell Immunotherapy for Relapsed B-cell Acute Lymphoblastic Leukaemia After CD19 CAR-T Adoptive Cellular Immunotherapy
NCT03464916	72	Active, not recruiting	Relapsed or Refractory Multiple Myeloma	CD38	Biological: CAR2 Anti-CD38 A2 CAR-T Cells	CAR2 Anti-CD38 A2 CAR-T Cells in patients with Relapsed or Refractory Multiple Myeloma
NCT03439280	100	Active, not recruiting	Relapsed/Refractory Multiple Myeloma	CD38	Drug: TAK-079 Drug: Pomalidomide Drug: Dexamethasone	TAK-079 Administered Subcutaneously as a Single Agent in Participants with Relapsed/Refractory (r/r) Multiple Myeloma (MM)
NCT03309111	197	Recruiting	Relapsed/Refractory Multiple Myeloma	CD38, CD3	Biological: ISB 1342	Single-Agent ISB 1342 in Subjects with Previously Treated Multiple Myeloma
NCT04000282	78	Recruiting	Plasma Cell Myeloma	CD38	Drug: SAR442085	SAR442085 in Patients with Relapsed or Refractory Multiple Myeloma (RRMM)
NCT01421186	91	Completed	Multiple Myeloma	CD38	Drug: MOR03087 phase 1 dose escalation Drug: MOR03087 Drug: Dexamethasone Drug: Pomalidomide Drug: Lenalidomide	Human Anti-CD 38 Antibody MOR03087 (MOR202) in Relapsed/Refractory Multiple Myeloma
NCT04466475	24	Not yet recruiting	Plasma Cell Myeloma	CD38	Biological: Astatine At 211 Anti-CD38 Monoclonal Antibody OKT10-B10 Drug: Melphalan Procedure: Peripheral Blood Stem Cell Transplantation	Anti-CD38 Monoclonal Antibody (211At-OKT10-B10) Combined with Melphalan as Conditioning Before Autologous Hematopoietic Cell Transplantation for Patients with Multiple Myeloma
NCT02136134	500	Active, not recruiting	Relapsed or Refractory Multiple Myeloma	CD38	Bortezomib and dexamethasone with or without daratumumab	An approach with the addition of daratumumab resulted in longer PFS and was linked with infusion-related reactions and higher rates of neutropenia and thrombocytopenia
NCT02076009	570	Active, not recruiting	Relapsed or Refractory Multiple Myeloma	CD38	Dexamethasone and lenalidomide with or without daratumumab	Adding daratumumab to the treatment increased PFS, the occurrence of infusion-related reactions, and a higher rate of neutropenia

**Table 5:** Daratumumab and Isatuximab in Lymphomas and Acute lymphoblastic leukaemia.

Identifier	Number of Patients	Status	Drugs Used	Disease	Short Description
NCT04251065	8	Active, not recruiting	Daratumumab, Gemcitabine, Dexamethasone, and Cisplatin	Relapsed or refractory T-Cell Lymphoma	
NCT04972942	40 (up to 39 years old)	Not yet recruiting	Daratumumab after total body irradiation (TBI)-based myeloablative conditioning and allogeneic hematopoietic cell transplantation	T-ALL	
NCT03432741	39	Recruiting	Direct tumor microinjection (into the skin or in lymph nodes) with drugs such as Belinostat, Carfilzomib, Copanlisib Hydrochloride, Daratumumab, Fludeoxyglucose F-18, Gemcitabine Hydrochloride, Nivolumab, Obinutuzumab, Pembrolizumab, Rituximab, Romidepsin. Trastuzumab. Patients will be also undergoing FDG-PET with	non-Hodgkin lymphoma, Hodgkin lymphoma,	
NCT03384654	47 (up to 30 years old)	Active, not recruiting	Daratumumab, vincristine, prednisone. Doxorubicin, Peg-asparaginase, cyclophosphamide, cytarabine, 6-mercaptopurine, methotrexate	B-cell ALL/LL, T-cell ALL/LL	
NCT04045028	60	recruiting	Tiragolumab, Daratumumab, Rituximab, Atezolizumab	Relapsed or Refractory B-Cell non-Hodgkin lymphoma	
NCT04136756	118	Recruiting	NKTR-225, NKTR-255 Q21, Rituximab, Daratumumab,	relapsed/refractory multiple myeloma and Non-Hodgkin’s Lymphoma, indolent Non-Hodgkin’s Lymphoma	
NCT01592370	316	Active, not recruiting	Nivolumab, Ipilimumab, Lirilumab, Pomalidomide, Dexamethasone. Daratumumab	Non-Hodgkin’s lymphoma, Hodgkin lymphoma	Nivolumab showed significant activity against B and T-cell lymphomas, while also being well-tolerated
NCT04017130	198	Recruiting	MT-0169	RRMM, RR NHL	
NCT03860844	96 (up to 17 years old)	Recruiting	Montelukast, Isatuximab, Dexamethasone, Fludarabine, Cytarabine, Liposomal daunonrubicin, daunorubicin, idarubicin, filgrastim, mitoxantrone. Doxorubicin, vincristine, PEG asparaginase, cyclophosphamide, etoposide, methotrexate, L-asparaginase, hydroxyurea, tocilizumab, L-asparaginase (erwinase)	Acute lymphoblastic leukaemia (ALL) and acute myeloblastic leukaemia (AML)	
NCT02999633	14	Terminated	Isatuximab, dexamethasone, acetaminophen, ranitidine, diphenhydramine	RR T-ALL or T-LL	The trial was terminated due to an especially high benefit/risk ratio.

**Table 6:** Daratumumab and Isatuximab (in combination with other chemotherapeutics) in Acute Myeloblastic Leukaemia.

Identifier	Number of Patients	Status	Drugs Used	Disease Treated
NCT04714372	50	Recruiting	Daratumumab, FT538, Fludarabine, Cyclophosphamide	AML
NCT02807558	155	Active, not recruiting	Tamibarotene, azacitidine, daratumumab	AML, Myelodysplastic syndrome
NCT04614636	105	Recruiting	FT538, Cyclophosphamide, Fludarabine, Daratumumab, Elotuzumab	AML
